# Resveratrol Butyrate Ester Supplementation Blunts the Development of Offspring Hypertension in a Maternal Di-2-ethylhexyl Phthalate Exposure Rat Model

**DOI:** 10.3390/nu15030697

**Published:** 2023-01-30

**Authors:** You-Lin Tain, Chih-Yao Hou, Guo-Ping Chang-Chien, Sufan Lin, Chien-Ning Hsu

**Affiliations:** 1 Department of Pediatrics, Kaohsiung Chang Gung Memorial Hospital, Kaohsiung 833, Taiwan; 2 College of Medicine, Chang Gung University, Taoyuan 330, Taiwan; 3 Department of Seafood Science, National Kaohsiung University of Science and Technology, Kaohsiung 811, Taiwan; 4 Institute of Environmental Toxin and Emerging-Contaminant, Cheng Shiu University, Kaohsiung 833, Taiwan; 5 Center for Environmental Toxin and Emerging-Contaminant Research, Cheng Shiu University, Kaohsiung 833, Taiwan; 6 Super Micro Mass Research and Technology Center, Cheng Shiu University, Kaohsiung 833, Taiwan; 7 Department of Pharmacy, Kaohsiung Chang Gung Memorial Hospital, Kaohsiung 833, Taiwan; 8 School of Pharmacy, Kaohsiung Medical University, Kaohsiung 807, Taiwan

**Keywords:** hypertension, developmental origins of health and disease (DOHaD), di-2-ethylhexylphthalate (DEHP), gut microbiota, butyrate, oxidative stress, resveratrol, short chain fatty acid

## Abstract

Resveratrol (REV) is a plant polyphenol with a plethora of beneficial properties. We previously enhanced the efficacy of REV via esterification of REV with butyrate to form resveratrol butyrate ester (RBE). Compared with REV, RBE exhibits higher bioavailability and better antioxidant effects. Hypertension can originate in early life because of maternal toxic chemical exposure. This study aims to examine the effectiveness of RBE in the protection of offspring hypertension induced by maternal di-2-ethylhexylphthalate (DEHP) exposure and to explore the underlying mechanisms. DEHP (10 mg/kg/day) was used as oral gavage to pregnant rats during gestation and lactation. The control group received the vehicle. Three groups of DEHP-exposed dams received REV (6.67 mg/kg/day), or low-dose (3.33 mg/kg/day) or high-dose (6.67 mg/kg/day) RBE in drinking water during gestation and lactation. Perinatal DEHP exposure resulted in hypertension and bodyweight gain in adult male offspring, which was prevented by high-dose RBE. REV supplementation attenuated DEHP exposure-induced increases in blood pressure but not bodyweight. High-dose RBE decreased renal oxidative damage, increased plasma butyrate concentrations, and altered short chain fatty acid receptor (SCFA) expression. Low-dose RBE treatment reduced downstream mediators of the acryl hydrocarbon receptor (AHR) signaling pathway. Moreover, DEHP exposure, REV and RBE treatment differentially shaped the offspring’s gut microbiota. In particular, high-dose RBE increased the abundance of the genus *Duncaniella*. The beneficial effects of RBE treatment were related to reducing oxidative damage, increasing plasma butyrate concentrations, downregulating SCFA receptor expression, antagonizing AHR signaling, and altering the gut microbiota. This study provides the first evidence of RBE as a novel plant polyphenol bioproduct targeting the oxidative stress and gut microbiota to protect against maternal DEHP exposure-primed offspring hypertension.

## 1. Introduction

Resveratrol is a plant polyphenol with a wide-ranging benefit on human health [1,2]. The antioxidant effects of resveratrol contribute substantially to its health benefits through scavenging reactive oxygen species (ROS), reducing oxidative stress and enhancing nitric oxide (NO) bioavailability [3,4]. Prior work indicates that resveratrol could have potential therapeutic and preventive value for hypertension [4,5]. We previously reported that resveratrol supplementation during gestation and lactation protected adult progeny against hypertension programmed by various maternal insults [6,7], which was attributed to its regulation on oxidative stress, the NO pathway, and aryl hydrocarbon receptor (AHR)-mediated renal inflammation.

In utero environmental chemical exposure can influence fetal development, resulting in hypertension in adult progeny [8]. In recent decades, the link between maternal exposure to environmental stimuli and the risk of developing adult disease has gained attention. This concept is often described as “developmental origins of health and disease” (DOHaD) [9]. Phthalates are typically added to plastics and used in plastic food wrap. Most humans are continuously exposed to phthalates due to their presence in food or in everyday consumer products [10]. Prior work demonstrated that phthalates in fatty meats, dairy products and processed foods are consistently found in high concentrations. Importantly, the exposure to phthalates may increase the risk of cardiovascular diseases [11]. Di-2-ethylhexylphthalate (DEHP) is the primary phthalate ester pollutant in the environment, and can cross the placenta and lead to adverse offspring outcomes [12]. DEHP exposure during pregnancy induced hypertension was observed in adult rat offspring [13].

The DOHaD concept offers an innovative approach to preventing hypertension via early-life intervention, the so-called reprogramming. Despite the fact that resveratrol can be used as a reprogramming therapy for the prevention of hypertension in different animal models of developmental origin [5], whether perinatal use of resveratrol can protect offspring against hypertension born to dams exposed to DEHP has not been fully elucidated. 

The poor bioavailability of resveratrol is a major concern for limiting its clinical translation [14]. Resveratrol is absorbed passively by diffusion or by forming complexes with intestinal membrane transporters. The gut microbiota is involved in the metabolism of resveratrol by increasing its availability from resveratrol precursors and producing resveratrol derivatives [15]. The major forms of resveratrol in the circulation are sulfate and glucuronide conjugate metabolites, leading to a very low level of its free form in the circulation [14]. 

The esterification of resveratrol could increase its absorption and assist its quick hydrolysis by an esterase inside cells [16]. In this regard, we previously enhanced the efficacy of resveratrol via the esterification of resveratrol with butyrate to form a resveratrol butyrate ester (RBE) [17]. Our prior work indicated that RBE contained RSV (~17.1%), RBE monoester (~47.1%), and RBE diester (~35.0%), which could effectively inhibit fatty acid-induced lipid accumulation and prevent chronic kidney disease-induced hypertension, with effects similar to those of RSV (50 mg/L), but achieved at a lower dose of RBE (25 mg/L) [18,19]. Hence, we aim to elucidate the effectiveness of RBE (at either low or high doses) in the protection of offspring hypertension induced by maternal DEHP exposure and elucidate the mechanisms involved in their beneficial effects.

## 2. Materials and Methods

### 2.1. Animal Model

All animal research was carried out with prior approval by our Institutional Animal Care and Use Committee (permit # 2021060802). Virgin Sprague Dawley (SD) rats were used at the beginning of the study (purchased from BioLASCO Taiwan Co., Ltd., New Taipei City, Taiwan). The study was performed in an AAALAC-accredited animal facility. Mating was achieved by placing one female and one male in a cage overnight. The presence of vaginal plugs was checked the next day for successful mating. 

**Figure 1:**
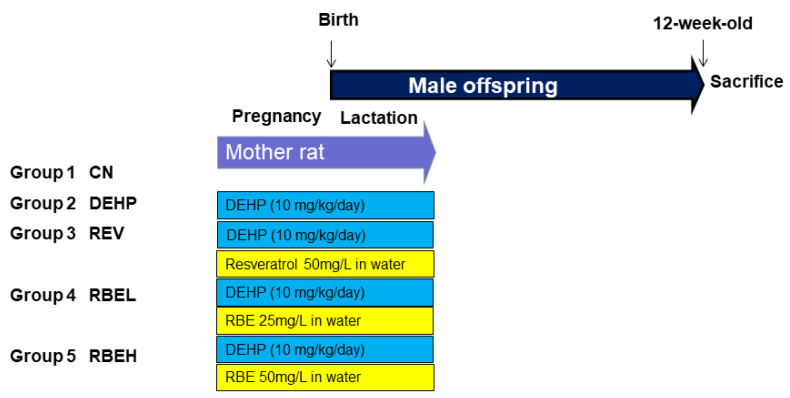
Experimental protocol used in the current study. CN = control; DEHP = Mother rats received di-2-ethylhexylphthalate (DEHP); REV = Mother rats received DEHP and resveratrol supplementation; RBEL = Mother rats received DEHP and low dose resveratrol butyrate ester (RBE); RBEH = Mother rats received DEHP and high dose RBE.

Experimental design is illustrated in Figure 1. Pregnant rats (n = 3/group) were assigned to five groups: control (CN), DEHP, DEHP + resveratrol (REV), DEHP + low dose RBE (RBEL), and DEHP + high dose RBE (RBEH). To conduct the DEHP exposure model, DEHP (10 mg/kg/day) or vehicle (corn oil) was oral gavage to pregnant rats during gestation and lactation. The dose of DEHP was chosen according to previous research [20]. Resveratrol (Sigma-Aldrich) was administered at the dose of 6.67 mg/kg/day in drinking water during gestation and lactation periods (for a total of 6 weeks). Resveratrol was prepared twice weekly by dissolving the drug in ethanol and then diluting with water to a final concentration of 50 mg/L. Water bottles were wrapped with aluminum as we published previously [6,7]. RBE was administered at the dose of 3.33 mg/kg/day (low dose) and 6.67 mg/kg/day (high dose) in drinking water during gestation and lactation, respectively. As RBE is a mixture of REV (~17.1%) and its derivatives (RBE monoester ~47.1%; RBE diester ~35.0%) [17,18], 50 mg/L of RBE is equivalent to the dose of 8.5 mg/L REV with 41.5 mg/L REV derivatives. After birth, litters were reduced to eight pups to standardize maternal pup care and quantity of milk. Considering that males develop hypertension earlier than females [21], we only selected male progeny for use in subsequent experiments. 

Blood pressure (BP) was measured at the age of 3, 4, 8, and 12 weeks using the CODA rat tail-cuff system (Kent Scientific Corporation, Torrington, CT, USA) [6,7]. One week before the actual recording sessions, the rats were adapted to restraint and tail-cuff inflation. At 12 weeks of age, all the rats were sacrificed. Fresh feces samples were collected and stored at −20 °C until extraction. The perfused kidneys were harvested, divided into the cortex and medulla, and stored at −80 °C until analyses. Blood samples were collected in heparinized tubes. The aliquot tubes were kept at −80 °C in a freezer.

### 2.2. Measurement of SCFAs by GC-MS

Gas chromatography (GC)-mass spectrometry (MS) (7890B, Agilent Technologies Santa Clara, CA, USA) equipped with an automated sampler was utilized to analyze plasma concentrations of SCFAs according to our previous protocol [22]. These SCFAs include acetic acid (C2), propionic acid (C3), butyric acid (C4), valeric acid (C5), and hexanoic acid (C6). Chromatographic separation was carried out by using a DB-FFAP column (30 cm × 0.25 mm, 0.25 µm; Agilent Technologies). The injection volume was 1 µL with a split ratio of 5:1 at 240 °C. We used 2-ethylbutiric acid as the internal standard. 

### 2.3. Measurement of NO Parameters by HPLC 

L-arginine is the substrate for NO synthase (NOS). Symmetric and asymmetric dimethylarginine (SDMA and ADMA) are both endogenous NOS inhibitors. These NO parameters in the plasma were determined using a high-performance liquid chromatography (HPLC) method (HP series 1100; Agilent Technologies Inc.) with fluorescence detection for o-phthalic aldehyde (OPA) derivatization with 3-mercaptopropionic acid (3MPA) [6]. Additionally, plasma creatinine concentrations were analyzed by HPLC [7].

### 2.4. Quantitative Real-Time Polymerase Chain Reaction

RNA was extracted from the kidney cortex of each rat. Renal gene expression of SCFA receptors and AHR signaling were analyzed by quantitative polymerase chain reaction (qPCR) using a SYBR Green PCR Reagents kit (Qiagen, Valencia, CA, USA); results were normalized to the 18S rRNA (R18S) reference gene as described [6,7]. Four SCFA receptors were analyzed, including G protein-coupled receptor 41 (GPR41), GPR43, GPR109A, and olfactory receptor 78 (Oflr78). Additionally, we analyzed the following AHR signaling pathway-related genes, including AHR, aryl hydrocarbon receptor repressor (AHRR), aryl hydrocarbon receptor nuclear translocator (ARNT), and cytochrome P450 CYP1A1 (CYP1A1). Each sample was run in duplicate and all qPCR reactions were followed by dissociation curve analysis. Table 1 illustrates the primer sequences of qPCR. Relative quantification of gene expression was performed using the comparative threshold cycle (Ct) method. We calculated the fold-increase in the target gene, relative to the reference gene, using formula 2^−ΔΔCt^.

### 2.5. Microbiota Sequencing

Microbial DNA was extracted from stool samples. The bacterial 16S rRNA gene was used for metagenomics analysis at the Biotools Co., Ltd. (New Taipei City, Taiwan) [20]. The full-length 16S genes were amplified using barcode primers adapted for SMRTbell library preparation and sequencing (PacBio, Menlo Park, CA, USA). All the downstream analyses on these sequences were carried out by using the QIIME2 software package [23]. A phylogenetic tree was constructed from the amplicon sequence variants (ASVs) via FastTree (QIIME2). The α-diversity indices, Shannon index and Faith’s phylogenetic diversity (PD) index were determined at ASV level. We examined 2 β-diversity indices, the analysis of similarities (ANOSIM), and the principal coordinate analysis (PCoA) of unweighted UniFrac distance to characterize the similarities between communities across groups. 

### 2.6. Immunohistochemical Detection of 8-OHdG 

8-Hydroxydeoxyguanosine (8-OHdG), a widely-used biomarker of oxidative DNA damage, was utilized to detect oxidative stress [24]. The kidney sections were deparaffinized with xylene and dehydrated through a graded ethanol series. After blocking with immunoblock (BIOTnA Biotech., Kaohsiung, Taiwan), the kidney sections were incubated with an anti-8-OHdG antibody (1:100, JaICA, Shizuoka, Japan) for 2 h. The immunohistochemistry was performed using the polymer-horseradish peroxidase detection system with 3,3′-diaminobenzidine (DAB) (BIOTnA Biotech). We used consecutive serial sections for IHC staining. Scoring for 8-OHdG-positive cells in high-power fields (200×) in the renal sections was carried out by counting the numbers from kidney sections as previously described [7]. All specimens were evaluated by 5 to 10 fields per case.

### 2.7. Statistics

All data are presented as means ± the standard error of the mean (SEM), and *p* < 0.05 was considered statistically significant. The data were subjected to a one-way analysis of variance (ANOVA). To produce post hoc multiple comparison tests, Tukey’s post hoc test was utilized. 

## 3. Results

### 3.1. Body Weight and Blood Pressure

**Figure 2:**
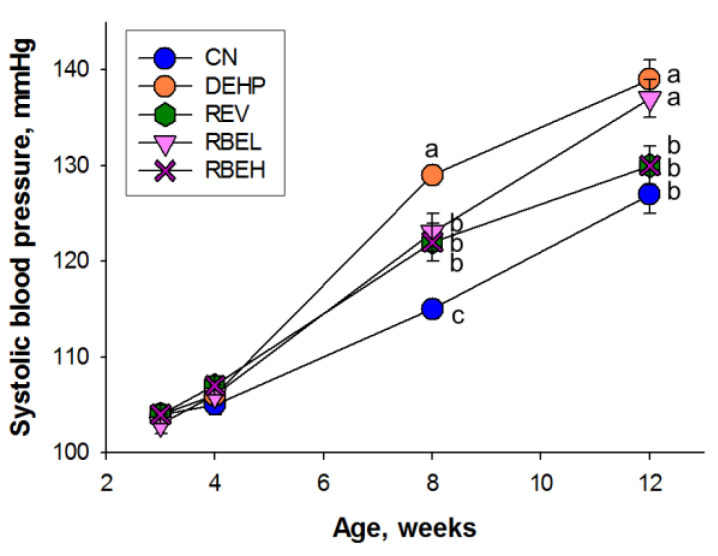
Effects of di-2-ethylhexylphthalate (DEHP), resveratrol (REV), and resveratrol butyrate ester (RBE) on systolic blood pressure in offspring from 3 to 12 weeks of age. The letters a, b and c indicate the differences between the groups (*p* < 0.05, two-way ANOVA with post hoc Tukey’s test); N = 8/group.

There were no deaths in any of the groups (Table 2). The bodyweight (BW) of the DEHP, REV and RBEL groups was higher than that in the CN and RBEH group. Maternal DEHP exposure caused a greater kidney weight (KW) than controls, which was prevented by both doses of RBE treatment. Also, maternal DEHP-exposed offspring receiving either a low-dose or high-dose of RBE exhibited a lower KW-to-BW ratio compared to that of the DEHP group. Figure 2 illustrates that systolic BP was increased in adult DEHP-exposed offspring at the age of 12 weeks that became significant at eight weeks of age. At 8 weeks of age, DEHP-induced increases in systolic BP were attenuated in the REV, RBEL and RBEH groups, while only high-dose RBE and REV treatments restored systolic BP and mean arterial pressure back to a normal range at 12 weeks of age (Table 2). 

### 3.2. Oxidative Stress and NO Parameters

**Figure 3:**
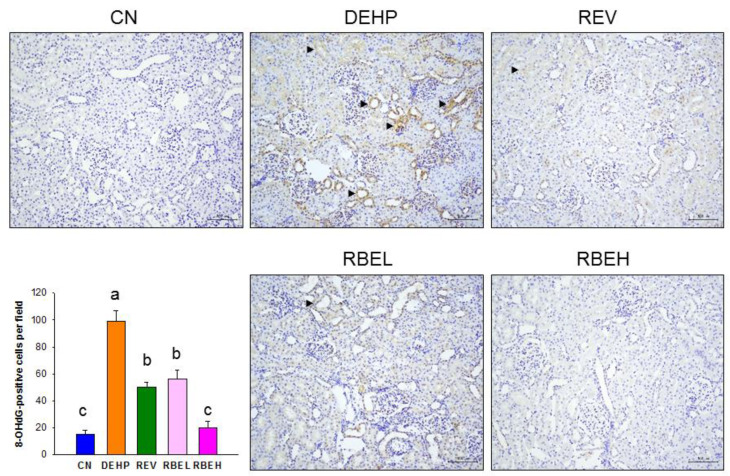
Light micrographs illustrating immunostaining for 8-OHdG in the 12-week-old male offspring kidney (200×). In the CN and RBEH group, 8-OHdG expression is almost negative. Immunostaining of 8-OHdG is at a moderate density in the glomeruli and tubules (arrowheads) in the REV and RBEL groups, and illustrating intense staining in the DEHP group. The letters a, b and c indicate the differences between the groups (*p* < 0.05, one-way ANOVA with post hoc Tukey’s test); N = 8/group.

The beneficial effects of resveratrol on programmed hypertension have been link to the reduction of oxidative stress and the enhancement of NO bioavailability [6]. We therefore investigated whether RBE has a protective role on DEHP-induced oxidative stress. We assessed oxidative damage in the offspring kidney by immunostaining 8-OHdG [24]. As shown in Figure 3, the intensity of cytosol and nuclear expression of 8-OHdG illustrated intense staining in the glomeruli and tubules of the DEHP group, an intermediate density of staining in the REV as well as the RBEL group, and little staining in the CN and RBEH group. 

**Figure 4:**
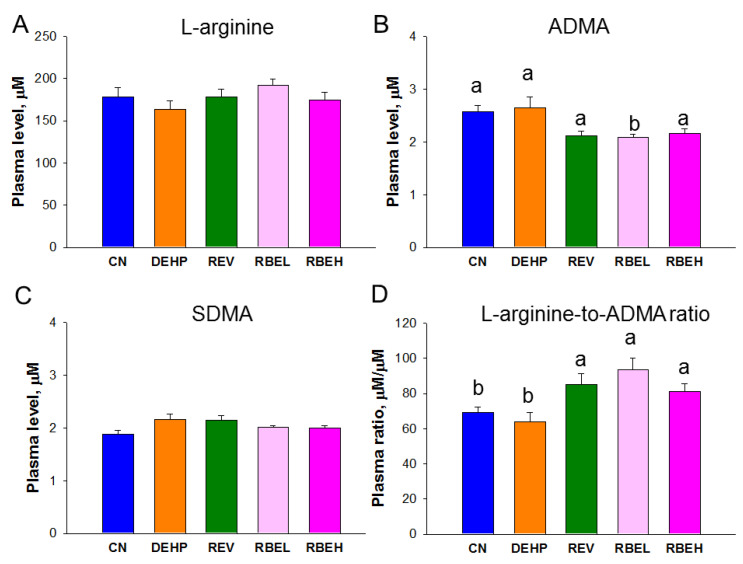
Effects of di-2-ethylhexylphthalate (DEHP), resveratrol (REV), and resveratrol butyrate ester (RBE) on plasma concentrations of (**A**) L-arginine, (**B**) asymmetric dimethylarginine (ADMA), (**C**) symmetric dimethylarginine (SDMA), and (**D**) the ratio of L-arginine-to-ADMA in offspring at 12 weeks of age. The letters a and b indicate the differences between the groups (*p* < 0.05, one-way ANOVA with post hoc Tukey’s test); N = 8/group.

Regarding the NO parameters, plasma concentrations of L-arginine and SDMA were not different among the five groups. As shown in Figure 4, the plasma concentration of ADMA was lowest in the RBEL group. Low-dose RBE treatment significantly increased NO bioavailability, represented by the ratio of L-arginine-to-ADMA, in the REV, RBEL and RBEH groups. 

### 3.3. Plasma SCFA Concentrations and Renal SCFA Receptors

We determined major SCFA concentrations in the plasma and their receptors in offspring kidneys. Table 3 shows that either low- or high-dose RBE caused an increase in plasma concentrations of butyric acid compared to the other three groups. The plasma hexanoic acid concentration was higher in the DEHP, REV and RBEL group than in the CN and RBEH groups. In addition, plasma concentrations of acetic acid, propionic acid and valeric acid had no differences among the five groups. 

**Figure 5:**
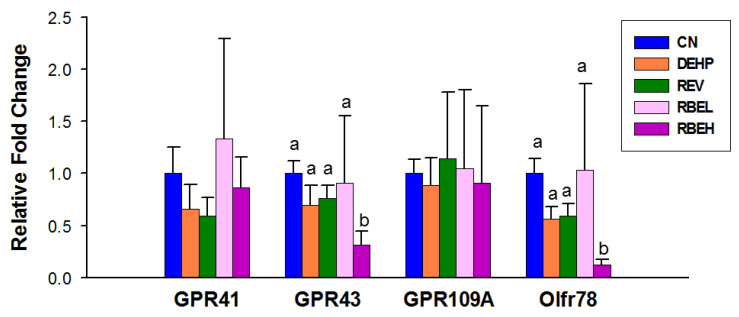
Effect of di-2-ethylhexylphthalate (DEHP), resveratrol (REV), and resveratrol butyrate ester (RBE) on short chain fatty acid (SCFA) receptors in 12-week-old male offspring kidneys, including G protein-coupled receptor 41 (GPR41), GPR43, GPR109A, and olfactory receptor 78 (Oflr78). The letters a and b indicate the differences between the groups (*p* < 0.05, one-way ANOVA with post hoc Tukey’s test); CN = control; N = 8/group.

We then analyzed the mRNA expression of SCFA receptors in offspring kidneys. Figure 5 illustrates that DEHP exposure had negligible effects on the renal mRNA expression of four SCFA receptors. However, high-dose RBE supplementation significantly reduced the renal expression of GPR43 and Olfr78 compared to the CN group. 

### 3.4. AHR Signaling

**Figure 6:**
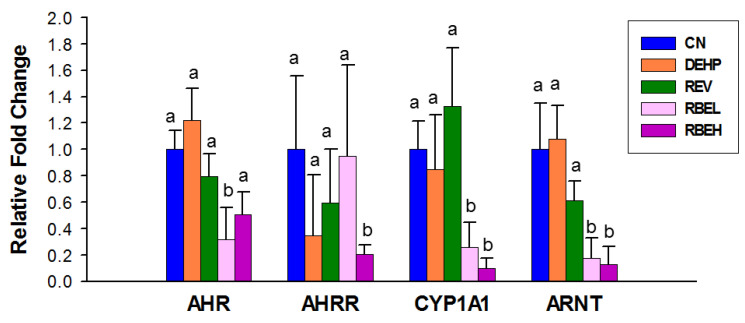
Effect of di-2-ethylhexylphthalate (DEHP), resveratrol (REV), and resveratrol butyrate ester (RBE) on the renal mRNA expression of the acryl hydrocarbon receptor (AHR) signaling pathway of male offspring at 12 weeks of age. AHRR= aryl hydrocarbon receptor repressor; CYP1A1 = cytochrome P450 CYP 1A1; ARNT = aryl hydrocarbon receptor nuclear translocator. The letters a and b indicate the differences between the groups (*p* < 0.05, one-way ANOVA with post hoc Tukey’s test); CN = control; N = 8/group.

We next determined AHR signaling, as resveratrol was beneficial for the control of AHR-related toxicity. Regarding the AHR signaling pathway, Figure 6 shows that the renal mRNA expression of AHR was lower in the RBEL group than that in the CN and DEHP groups. Additionally, low-dose RBE treatment significantly reduced the renal mRNA expression of CYP1A1 and ARNT. Similarly, the renal mRNA expression of CYP1A1 and ARNT were lower in the RBEH group compared to the CN and DEHP groups. 

### 3.5. Differences in Microbiota Compositions

**Figure 7:**
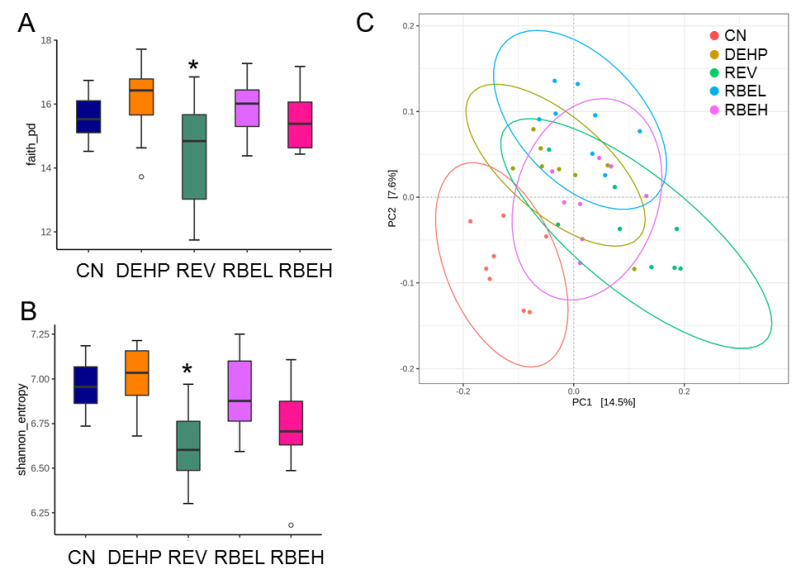
Bacterial α-diversity for gut microbial communities among five groups in (**A**) Faith’s phylogenetic diversity (PD) index and (**B**) the Shannon index; ht panel). The outliers are shown as dots. * *p* < 0.05. (**C**) Bacterial β-diversity analysis using the principal coordinate analysis (PCoA) based on unweighted UniFrac distance of the OTUs in five groups; each dot represents the microbiota of a single sample, and the color of the dot reflects the metadata for that sample. N = 8/group.

Figure 7 illustrates that REV treatment reduced community richness and evenness, represented by the Faith’s PD index and the Shannon index, in the REV group compared with those in the CN group. To examine bacterial community structural differences among five groups (i.e., β-diversity), a PCoA based on unweighted UniFrac metric indicated that the microbial communities from the five groups were completely differentiated into different clusters (Figure 7C). Furthermore, the overall difference between grouped communities can be judged by ANOSIM. The ANOSIM test did yield statistically significant differences among the groups. We observed that every group differed from each other (All *p* < 0.01). 

**Figure 8:**
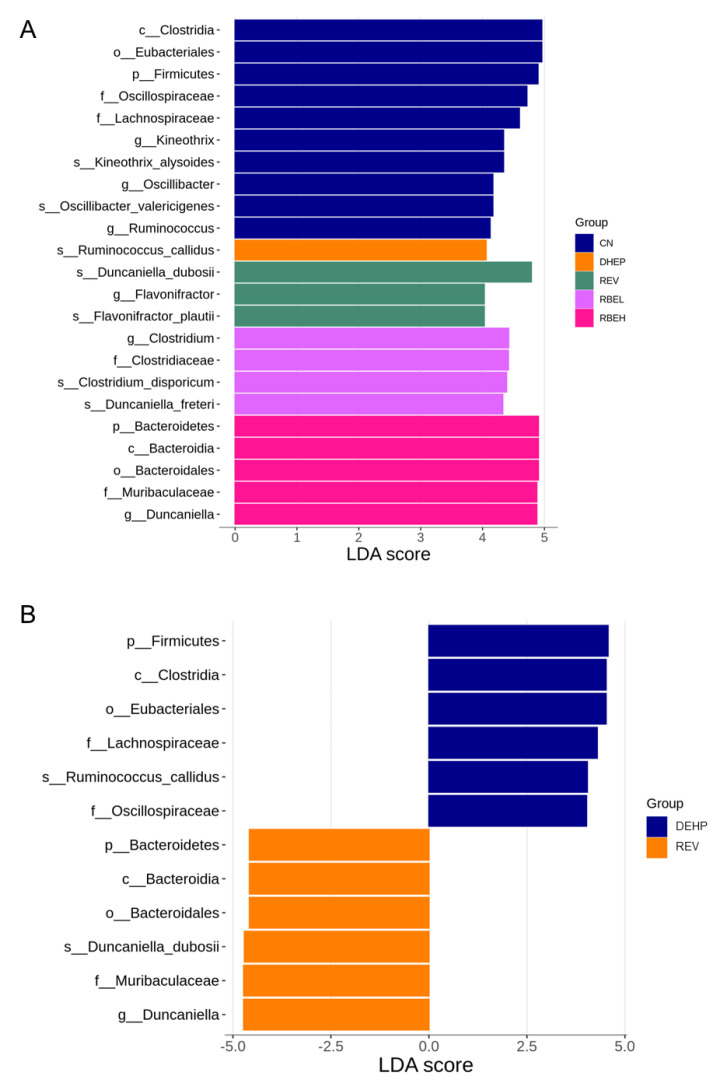

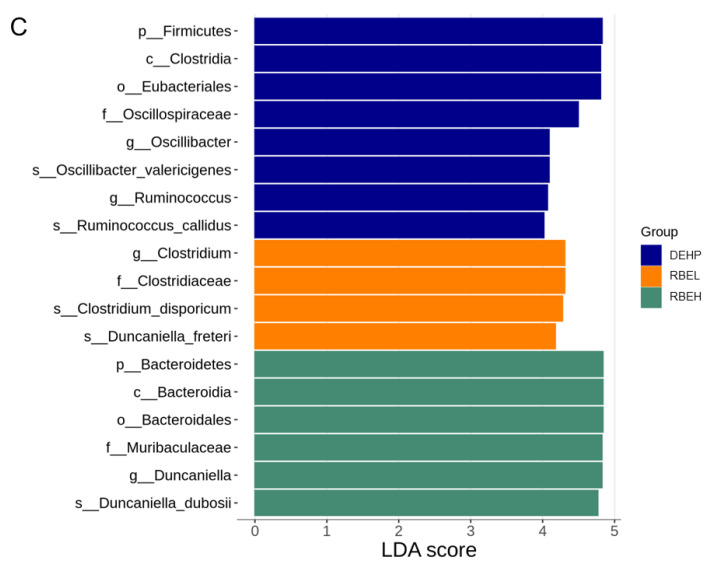
Linear discriminant analysis effect size (LEfSe) comparing the differentially abundant taxa between (**A**) all five groups; (**B**) the DEHP and REV group; and (**C**) the DEHP, RBEL, and RBEH groups. The linear discriminant analysis (LDA) score threshold was set to greater than 4.

A linear discriminant analysis effect size (LEfSe) was performed, allowing for the comparison of taxonomies considered significantly differentially abundant among the five groups (Figure 8A). A total of 12 taxa exhibited significantly different abundances in the comparison between the REV and DEHP groups (Figure 8B). Specifically, the genus *Duncaniella* and the family, order, class, and phylum to which it belongs was more abundant in the REV group. Similarly, high-dose RBE treatment caused a higher proportion of genus *Duncaniella* and the family, order, class and phylum to which it belongs (Figure 8C). As a result, low-dose RBE treatment resulted in an increase in the genus abundance of *Clostridium*, a butyrate-producing bacteria, in the RBEL group compared with the DEHP group (Figure 8C).

## 4. Discussion

In the present study, we established a maternal DEHP exposure model to induce offspring hypertension and investigated the protective effects of RBE. Our most noteworthy findings include: (1) DEHP exposure during gestation and lactation causes hypertension and BW gain in adult male offspring, which high-dose RBE prevents; (2) high-dose RBE and REV similarly protected against maternal DEHP exposure-induced offspring hypertension; (3) DEHP-primed offspring hypertension is associated with kidney oxidative damage characterized as increased 8-OHdG staining; (4) high-dose RBE averts adult progeny against hypertension accompanied by reducing oxidative damage, increasing plasma butyrate concentrations, and altering SCFA receptor expression; (5) DEHP exposure, REV and RBE treatment differentially shaped the offspring’s gut microbiota; and (6) high-dose RBE and REV both caused the increases in the abundance of the *Duncaniella* genus. 

The association between DEHP exposure and hypertension has been recognized [8,10,13], although evidence for the underlying mechanism in maternal DEHP exposure-induced offspring hypertension is wanting [13]. Our current study demonstrated, for the first time, that DEHP exposure during gestation and lactation induced hypertension and obesity in adult male offspring, which was prevented by high-dose RBE. Although REV is as effective as high-dose RBE on DEHP-primed offspring hypertension, it exhibits a neglectable effect on BW gain. Compared with high-dose RBE, the BP-lowering effect of low dose RBE cannot persist until the offspring at 12 weeks of age. Our results fit in with previous studies showing that maternal DEHP exposure leads to adverse offspring outcomes, including hypertension and obesity [13,25]. 

Our prior research indicates that maternal chemical exposure gives rise to oxidative stress-related renal programming and offspring hypertension in the presence of increased renal 8-OHdG expression [26,27]. In view of the fact that DEHP-primed offspring hypertension was accompanied by increased 8-OHdG staining, our study supports the notion that oxidative stress might be one major mechanism behind hypertension induced by DEHP exposure. 

In support of the antioxidant properties of RBE and REV [1,2,3,14], the treatment of maternal rats with RBE or REV prevents offspring hypertension coinciding with the reduction of oxidative damage. Low-dose RBE treatment and REV similarly attenuated renal oxidative damage in adult offspring born to dams exposed to DEHP. Notably, only high-dose RBE treatment is able to restore oxidative damage and BP completely, suggesting that the protective effects of RBE against oxidative stress might be in a dose dependent manner. Although prior work demonstrates that REV protects adult progeny against hypertension and is associated with the restoration of NO bioavailability [6,28], our study failed to identify the impact of high-dose RBE or REV on the NO pathway. 

Another positive effect of RBE against DEHP-induced hypertension might involve its ability to increase butyrate concentration. Our data is consistent with previous work showing that supplementation with butyrate esters can result in an increase in plasma butyrate concentration [29]. Additionally, another possibility would be low-dose RBE treatment augmenting the abundance of the genus *Clostridium*, one of the common butyrate-producing bacteria. SCFAs can interact with their receptors to regulate BP [30]. The activation of GPR 41 and GPR109A can lower BP and can be counteracted by GPR43 and Olfr78 to induce vasoconstriction. High-dose RBE reduced GPR 43 and Olfr78 expression would, therefore, have a vasodilatory effect and lower BP. Accordingly, the overall effects of RBE on butyrate production and its actions on SCFA receptors could give rise to its benefit against offspring hypertension in this model.

Another beneficial effect of RBE treatment might be due to its ability to antagonize AHR-mediated gene transcription. Previous research reports that the AHR/ARNT signaling pathway is a target for the prevention of DEHP-induced toxicity, and resveratrol is a natural AHR antagonist [31,32]. We observed that high-dose RBE that protected offspring against hypertension coincided with decreased renal expression of ARNT and AHR downstream of the target gene CYP1A1. Considering that the activation of the AHR/CYP1A1 axis can induce vasoconstriction [33], RBE suppressed renal ARNT/CYP1A1 expression might, at least in part, contribute to its beneficial actions against DEHP-primed hypertension.

Our data presented that the beneficial effects of RBE are also linked to alterations of the gut microbiota. Prior research indicates that *Duncaniella*, a butyrate enriched species, contributes to the colitis protection with anti-inflammatory properties [34]. We observed that maternal high-dose RBE and REV treatments both increased the abundance of the genus *Duncaniella*. According to our data, high-dose RBE protected offspring hypertension accompanied by increased butyrate and its enriched genus *Duncaniella*. Our former work indicated that perinatal butyrate supplementation protected adult offspring against hypertension induced by a maternal tryptophan-free diet [35]. Thus, further studies linking the abundance of *Duncaniell* and butyrate production behind the BP-lowering effect of RBE and REV are still required. Furthermore, additional studies are needed to elucidate whether the genus *Duncaniella* may serve as microbial markers for hypertension in other models of developmental programming. Despite the fact that low-dose RBE treatment increased butyrate and the butyrate-producing bacteria *Clostridium*, its BP-lowering effect was inferior to high-dose RBE. Moreover, our results contradict previous reports showing that REV increased α-diversity and prevented maternal CKD-primed offspring hypertension concurrently [28], perhaps since we were experimenting in a model of environmental toxin-induced developmental programming, which might exhibit a totally different mechanism. Taken together, our results showed that maternal RBE and REV differentially shifted offspring gut microbiota and altered derived metabolites, by which they provided protection against hypertension.

Our study also had limitations. Firstly, this is a male-only study. Whether sex differences exist in the preventive response of RBE remains unclear. Secondly, we only compared two testing doses of RBE with REV in the current study. Additional studies with multiple test doses of RBE for studying dose-dependent effects are still required. Thirdly, we mainly focused on the kidney in the present study. DEHP may cause various organ dysfunctions, leading to obesity, diabetes, fatty liver, etc. [12]. Our data indicated that maternal DEHP exposure caused body weight gain in adult offspring. Considering that DEHP, being exogenous compounds, primarily attack the liver, the protective effect of RBE against obesity may be attributed to the liver and other organs and is worthy of further evaluation. Whether body weight gain in DEHP-treated offspring is related to changes in food consumption or physical activity awaits further clarification. Lastly, we determined intestinal microbiota mainly in adult progeny at the time hypertension was happening, but not in mother rats. Studying the gut microbiota and the underlying mechanisms in dams and their offspring might offer more information on whether RBE and REV treatment either similarly or differentially shift the gut microbiota in dams and offspring, and whether maternal gut microbiota is linked to offspring outcome. 

## 5. Conclusions

In conclusion, our results demonstrate that an RBE supplement during gestation and lactation could protect offspring hypertension subjected to maternal DEHP exposure. The beneficial effects are mediated by reducing oxidative stress, shifting gut microbiota, rectifying the dysfunctional gut-kidney axis via targeting the microbial metabolite butyrate and SCFA receptors, as well as antagonizing AHR signaling in the offspring kidneys. Our data not only reinforces our understanding of mechanisms behind maternal DEHP exposure-primed offspring hypertension, but also provides the impetus to consider plant polyphenol bioproducts as a promising preventive approach for the maternal toxic chemical exposure-induced offspring hypertension.

## Figures and Tables

**Table 1:** PCR primer sequences.

Gene	Gene Accession No.	Forward	Reverse
GPR41	NM_001108912	5 tcttcaccaccgtctatctcac 3	5 cacaagtcctgccaccctc 3
GPR43	NM_001005877	5 ctgcctgggatcgtctgtg 3	5 cataccctcggccttctgg 3
GPR109A	NM_181476	5 cggtggtctactatttctcc 3	5 cccctggaatacttctgatt 3
Olfr78	NM_001000624	5 gaggaagctcacttttggtttgg 3	5 cagcttcaatgtccttgtcacag 3
AHR	NM_001308254	5 gtcctcagcaggaacgaaag 3	5 ccagggaagtccaactgtgt 3
AHRR	NM_001024285	5 cagcaacatggcttctttca 3	5 tgaagcactgcattccagac 3
ARNT	NM_012780	5 gtctccctcccagatgatga 3	5 gctggtagccaacagtagcc 3
CYP1A1	NM_012540	5 gcactctggacaaacacctg 3	5 atatccaccttctcgcctgg 3
R18S	X01117	5 gccgcggtaattccagctcca 3	5 cccgcccgctcccaagatc 3

**Table 2:** Weights and blood pressures of male offspring at 12 weeks of age.

Groups	CN	DEHP	REV	RBEL	RBEH
Body weight (BW) (g)	219 ± 10 ^b^	327 ± 16 ^a^	285 ± 15 ^a^	299 ± 6 ^a^	254 ± 7 ^b^
Left kidney weight (g)	1.21 ± 0.06 ^b^	1.7 ± 0.09 ^a^	1.46 ± 0.1 ^a^	1.4 ± 0.02 ^b^	1.19 ± 0.03 ^b^
Left kidney weight/100 g BW	0.55 ± 0.01 ^a^	0.52 ± 0.02 ^a^	0.51 ± 0.03 ^a^	0.47 ± 0.01 ^b^	0.47 ± 0.01 ^b^
Diastolic BP (mmHg)	84 ± 1	88 ± 5	85 ± 1	90 ± 2	85 ± 1
MAP (mmHg)	99 ± 1 ^b^	105 ± 4 ^a^	100 ± 1 ^b^	108 ± 2 ^a^	100 ± 1 ^b^

The superscripts a and b indicate the differences between the groups (*p* < 0.05, one-way ANOVA with post hoc Tukey’s test); N = 8/group; BP = blood pressure. MAP = mean arterial pressure.

**Table 3:** Plasma concentrations of SCFAs of male offspring at 12 weeks of age.

Groups	CN	DEHP	REV	RBEL	RBEH
Acetic acid, ng/mL	787 ± 124	770 ± 47	780 ± 72	1002 ± 52	819 ± 62
Propionic acid, ng/mL	7.0 ± 0.84	6.19 ± 0.41	6.43 ± 0.38	6.59 ± 0.57	6.58 ± 0.62
Butyric acid, ng/mL	3.12 ± 0.3 ^b^	2.47 ± 0.24 ^b^	2.26 ± 0.22 ^b^	6.38 ± 0.49 ^a^	7.1 ± 0.41 ^a^
Valeric acid, ng/mL	13.6 ± 0.32	0.97 ± 0.22	1.02 ± 0.21	1.23 ± 0.28	1.24 ± 0.23
Hexanoic acid, ng/mL	6.14 ± 0.35 ^b^	7.82 ± 0.35 ^a^	6.35 ± 0.37 ^a^	6.84 ± 0.54 ^a^	6.16 ± 0.35 ^b^

The superscripts a and b indicate the differences between the groups (*p* < 0.05, one-way ANOVA with post hoc Tukey’s test); N = 8/group.

## Data Availability

Data are contained within the article.
